# Supplementary material for: Are carbon emissions trading and green financial instruments synergistic? -Comprehensive quantitative research based on content analysis
Source: PLoS One. 2024 Mar 7;19(3):e0298601. doi: 10.1371/journal.pone.0298601 (PMC10919601; doi:10.1371/journal.pone.0298601)
Supplement: S1 Data — (ZIP) [file pone.0298601.s001.zip › Data/地级市Z_F工作报告.zip!/░▓╗╒/╗╞╔╜╩╨/└┤╫╘╣1⁄2╓┌║┼í╛┬φ┐╦╩2╛▌═°í┐_2018.docx]

各位代表：

现在，我代表市人民政府，向大会报告政府工作，请予审议，并请市政协委员和其他列席人员提出意见。

**风雨兼程，砥砺奋进——过去五年工作回顾**

过去的五年，是黄山发展进程中极不平凡的五年。五年来，面对复杂多变的宏观环境和艰巨繁重的改革发展任务，全市上下在省委、省政府和市委的坚强领导下，全面贯彻落实党的十八大和十九大精神，坚持以新发展理念为引领，坚持稳中求进工作总基调，扎实推进供给侧结构性改革，全力实施五大发展行动计划，统筹推进稳增长、促改革、调结构、惠民生、防风险各项工作，迎难而上，开拓进取，全市经济持续健康发展，人民生活显著改善，社会大局和谐稳定，较好地完成了市六届人大历次会议确定的目标任务，为全面建成小康社会奠定了坚实基础。

**——主要指标迈上新台阶。**全市生产总值连续跨上500亿元、600亿元两个台阶，人均生产总值突破6000美元关口；财政收入跨越100亿元；固定资产投资突破600亿元；社会消费品零售总额超300亿元；游客接待量超5700万人次，旅游总收入突破500亿元，标志我市经济发展水平抵达一个新的发展高度，展现出良好的韧性和活力。

**——现代综合交通体系基本建成。**合福高铁通车运营，杭黄高铁联调联试，池黄高铁、昌景黄高铁前期工作加快推进，黄山全面进入高铁时代；黄祁高速建成通车，芜黄高速开工建设，黄千高速、德上高速即将开工，黄山机场迁建、黄山旅游轻轨等重大项目取得进展，全市高速公路密度达到长三角发达地区平均水平，黄山连南通北、承东启西的区域综合交通枢纽地位日益凸显。

**——城乡基础设施建设取得突破。**皖南现代化区域性中心城市加快建设，建成区面积扩大到60平方公里，城市道路通达性和路网完整性持续提升，公共交通体系不断健全，自来水普及率、污水集中处理率和城市绿化率居全省前列。农村基础设施大幅改善，农村道路、农网供电、安全饮水等公共服务水平不断提高。我市历史上最大的水利枢纽工程——月潭水库开工建设，黄山人民半个世纪的梦想成为现实。

**——特色小镇和美丽乡村建设走在全省前列。**在全省率先启动特色小镇建设，东黄山国际小镇建设全面拉开，宏村镇、齐云山镇获批“中国特色小镇”，西溪南创意小镇、齐云旅游小镇和太平湖运动休闲小镇跻身首批省级特色小镇。打造具有黄山特色的美丽乡村“升级版”，在全省连续3轮考核中位居前列，屯溪区、黄山区、徽州区、歙县荣获全省美丽乡村建设先进区县称号，皖风徽韵的美丽乡村更加彰显。

**——生态环境建设成效卓著。**完成全国首个跨省流域新安江生态补偿机制两轮试点，举办首届新安江绿色发展论坛，设立新安江绿色发展基金，在全省率先建成农药集中配送体系，补偿机制试点工作入选2015年全国十大改革案例，为国家建立完善生态保护补偿机制提供了示范。全市森林覆盖率82.9%，地表水水质优良率、集中式饮用水水源地水质达标率均为100%，空气质量优良率常年保持在95%以上,生态环境质量保持全国领先。

**——人民群众获得感、幸福感、安全感显著增强。**五年累计安排民生类资金635亿元，是前五年的2.3倍。城镇常住居民人均可支配收入30810元，农村常住居民人均可支配收入14030元，农村居民人均住户存款余额居全省前列。脱贫攻坚成效显著，153个贫困村全部出列，115296名贫困人口稳定脱贫，贫困发生率从2012年底的10.9%下降至2017年底的1.21%。我市蝉联“长安杯”，黄山风景区在全国景区中首捧“长安杯”，成为全国唯一拥有两个“长安杯”的城市，群众安全感位居全省前列。

五年来，我们主要做了以下工作：

**一是坚持不懈稳增长、促发展，综合实力较快提升。**加大对实体经济支持力度，全面落实国家和省各项政策措施，聚焦突出问题，聚焦企业需求，制定出台并动态调整促进重点产业发展“1+N”政策体系，落实市级领导联系重点企业、重点项目等制度，扎实开展“四送一服”双千工程。五年累计安排扶持实体经济发展财政资金15.7亿元，解决企业过桥贷款76.8亿元、税融通贷款10.5亿元，融资担保贷款余额49.5亿元，小微企业贷款五年净增199亿元，2017年新增贷款首次突破百亿。积极对接省五大发展行动计划和“三重一创”建设，谋划储备重点项目760个、总投资超3000亿元，一批重大项目纳入国家和省“十三五”规划，五年累计争取中央和省补助资金43.5亿元，国家专项建设基金37.4亿元。严格落实“四督四保”制度，组织开展重点项目“百日会战”“百日攻坚”“双百会战”，每年滚动推进300个左右市级重点项目，中建材凯盛新材料、富田精工、盛锐精工、康师傅矿泉水等项目建成运营，歙黟一级公路、小罐茶、黄山电器新型器件研发、黄山博蓝特半导体等项目快速推进，五年累计完成重点项目建设投资1847亿元，为黄山可持续发展打下了坚实基础。

**二是坚持不懈转方式、调结构，产业层次稳步提高。**着力“做精一产、做强二产、做优三产”，三次产业结构由2012年的11.4∶46.3∶42.3调整为2017年的9∶40.2∶50.8。大力发展精致农业，规上农产品加工企业230家，产值240亿元，年均增长9.9%；茶业综合产值达132亿元，茶叶出口额占全国12%、全省70%以上，国家茶叶及农产品检测重点实验室建成使用，我市被授予“中国名茶之都”；特色产业基地超170万亩，休宁山泉流水养鱼和太平猴魁茶文化系统入选中国重要农业文化遗产；黟县“有农模式”积极复制推广；电子商务进农村实现全覆盖；成功举办中国休闲农业黄山峰会、中国茶叶经济年会，黄山区、休宁县分别跻身国家现代农业示范区、全国农业产业化示范基地。大力发展新型工业，制定实施《黄山市工业转型升级规划》，聚力培育主导产业、市级队企业，建成省级新型工业化产业示范基地4个、产业集群专业镇15个，净增规上工业企业136户，规上工业增加值年均增长9.6%；战略性新兴产业产值年均增长12.2%；建成国家级创新平台7个、国家级企业孵化器1家、院士工作站7家、博士后工作站14家；新增国家知识产权示范企业2家，高新技术产业增加值年均增长11.9%；新增中国驰名商标11件，地理标志保护产品8个；“新三板”挂牌企业10家、省股权交易托管中心挂牌企业119家，全市直接融资216.5亿元；全市开发区规上工业总产值年均增长13.8%，市经济开发区在全省开发区综合考核中首次进入前30名。大力发展现代服务业，完成国家服务业综合改革试点任务，黄山现代服务业产业园入选省级战略性新兴产业集聚发展基地，全市服务业增加值年均增长9.6%。大力实施“旅游+”战略，勇闯“中国旅游，黄山再出发”新路，聚焦“四全黄山”，把全域旅游作为主攻方向，深入推进皖南国际文化旅游示范区“1+5”行动计划，着力打造世界一流旅游目的地，全市游客接待量、旅游总收入年均分别增长9.7%、10.8%，入境游客238万人次，过夜登记游客突破900万人；古徽州文化旅游区晋级国家5A景区，黄山风景区荣获全球首个世界优秀目的地景区，黄山旅游集团公司连续九年跻身中国旅游集团20强。

**三是坚持不懈抓改革、扩开放，发展活力日益增强。**深入实施“放管服”改革，大力推行“互联网+政务服务”，建立健全“3+2”清单体系，市级行政权力事项和行政审批项目分别精简70.7%、52%，非行政许可审批全部取消。深化商事制度改革，全面施行“多证合一、一照一码”，五年净增各类市场主体4.67万户。在全国率先探索开展旅游管理领域相对集中行政处罚权试点工作，在全省创新设立旅游仲裁院和旅游警务支队，实现旅游市场监管、执法、查处“三统一”。城管执法体制改革试点基本完成。农村土地承包经营权、集体土地确权登记颁证工作全面完成，黄山区农村承包土地经营权抵押贷款试点有序推进，黟县农业保险试点经验在全省推广。江南林交所业务网络实现全省覆盖。不动产统一登记制度全面实施。全市统一规范开放的公共资源交易市场基本建成。市属53家事业单位“撤并转”改革有序实施。黄山旅游集团、城投集团、文投集团、信保集团、供销集团改革转型基本完成。发起设立天使投资基金、创业投资基金和股权投资基金，农村互助金融走在全国前列。积极参与“锦绣安徽·迎客天下”全球推介，承办中俄总理定期会晤委员会双方主席会晤等重大活动，举办黄山旅游节、联合国教科文组织黄山对话会、杭州G20畅游黄山等活动。深化与德国斯特拉尔松德市、瑞典瓦拉市和瑞士因特拉肯市等友好城市交往，与俄罗斯斯捷尔利塔马克市、英国高峰市、西班牙赫罗纳市等结成友好交流城市，黄山海峡两岸交流基地获批设立，我市成为加入中俄蒙“万里茶道”国际旅游联盟的唯一地级市。黄山屯溪机场升格为国际机场，黄山航空口岸对外国籍飞机开放，台湾居民口岸办证和外国人口岸签证业务开通，口岸出入境突破10万人次。强力推进招商引资，组建市级专业小分队开展驻点招商，依托“商会合作共建皖江——黄山行”等活动平台，大力开展以商招商和产业链招商。与北京市国资委及北京城建等市属企业签署战略合作协议，联合利华、科大讯飞、深圳益田、广安控股、北京陶冶等企业落户我市，累计到位内资1703亿元，其中亿元以上项目632个。

**四是坚持不懈强基础、提品位，城乡面貌焕然一新。**大力实施全国“城市双修”试点和“两治三改”专项行动，五年完成城镇固定资产投资2230亿元，是前五年的2倍，户籍人口城镇化率达33%。全面加快中心城区“一环三片”建设，东外环、梅林南路、文峰桥建成通车，高铁客运枢纽、迎宾大道综合改造及地下管廊投入使用，江南新城建设快速推进。黎阳in巷、黄山银街、黄山农产品物流园（一期）建成运营，屯溪老街滨江西路改造提升全面实施。稳步推进人行道及绿化提升工程，新建改造道路32条、公园广场13座，建成运营公共租赁自行车、共享汽车项目，全面完成农贸市场改造。市城市展示馆、图书馆建成使用，公共停车场、生活垃圾综合处理厂等项目快速推进。强力推进市域南部城镇群和区县城建设，黄山区环太平湖道路网、徽州区岩寺老街暨丰乐河综合整治、歙县城建“八大工程”、休宁县“六纵五横”城市道路、黟县古城“九街十三巷”修缮改造、祁门县城区三大组团等一批项目基本建成。推进全域环境整治常态化、长效化，组织开展“三线三边”环境整治、农村环境“三大革命”，启动实施全市农村生活垃圾、污水治理PPP项目，推广休宁流口“垃圾兑换超市”，全力推进世行贷款黄山新农村建设项目，累计完成美丽乡村建设投资53.3亿元。在全省率先启动美丽社区建设，改造老旧小区171个，居民生活环境明显改观。城市数据中心、旅游大数据中心、社会服务管理信息化平台、数字城管平台建成运行，城市管理数字化、精细化水平全面提升。

**五是坚持不懈重保护、促利用，生态文化优势彰显。**扎实开展国家生态文明先行示范区建设，着力打造生态文明建设安徽样板的示范先行区。严格落实党政领导干部生态环境损害责任追究机制，认真抓好中央和省环保督察反馈问题整改落实，全面完成禁养区173家规模养殖场（户）关闭搬迁。覆盖全市的四级河长制全面建立。完成永久基本农田划定和土地利用总体规划调整完善工作。全面实施林长制，完成千万亩森林增长工程，开展林业增绿增效行动，大力实施松材线虫病三道防线工程，松材线虫病防治项目（二期）通过验收，荣获国家森林城市称号。徽州古建筑保护利用工程累计完成投资90.3亿元，建成一批乡贤好人馆、村史馆，打造一批文化客栈、书吧等新型业态。新增世界非遗子项目1个、国保单位14处，徽州府衙修复工程获“鲁班奖”。故宫博物院驻安徽黄山徽派传统工艺工作站、故宫学院黄山分院、故宫博物院博士后工作站成立，成功举办承办中国非物质文化遗产传统技艺大展、“徽匠神韵”故宫特展、“明月清风”故宫博物院藏新安八家书画展、“文化徽州高峰对话”。《曙光曲》等5部作品获省“五个一工程”奖。在全省率先完成二轮修志任务。《徽州文化大辞典》出版，《阅读徽州》被评为十佳皖版图书，徽州文化博物馆在全省地级市首家获评国家一级馆。建成一批国家级、省级文化产业基地和园区，“竹艺轩”列入国家级非遗生产性保护示范基地。我市入选全国首批十大历史建筑保护利用试点城市，获评“中国文房四宝文化名城”和“中国摄影之乡”。

**六是坚持不懈惠民生、增福祉，人民生活持续改善。**滚动实施54项省、市民生工程，累计完成投资218.5亿元，民生工程工作走在全省前列。城镇新增就业10.86万人，城镇登记失业率控制在3.8%以内。五项社会保险参保人数94.94万人次。企业退休人员退休费较2012年人均月增加683元，农村低保、分散供养五保、集中供养五保标准分别提高113%、178%、122%。在全省率先实现跨省异地就医结算，新农合参合率100%，累计发放城乡大病救助资金11.6亿元。开工建设公租房和棚户区改造住房30629套，累计建成保障房和棚户区改造住房56406套，向48286户发放住房租赁补贴9912万元，城镇中等偏下收入和低收入家庭住房保障实现应保尽保。扎实推进精准扶贫、精准脱贫，认真开展“万名党员干部帮万户”“百企帮百村”行动，深入实施脱贫攻坚“十大工程”，贫困地区生活生产条件明显改善。扎实推进地质灾害隐患点治理，3092户10796人消除了地质灾害威胁。制定出台“人才10条”“技能人才15条”，建立人才储备金制度，引进高层次人才100余名，培养高技能人才1.85万人，孙伟、汪仕洋在第44届世界技能大赛上夺取铜牌，实现我省奖牌“零”的突破。全市万人发明专利拥有量4.8件。调整优化中心城区教育布局，完成屯溪一中等三校联动搬迁，恢复设立屯溪三中，整合设立黄山职业技术学院和炎培职业学校，挂牌成立黄山职业技术学院国际护理学院，县区全部通过“义务教育发展基本均衡县”国家认定。全面深化综合医疗体制改革，城乡居民免费享受11项重大公共卫生服务和14类基本公共卫生服务；市医院综合大楼建成，市中医院、市二院、市疾控中心投入使用，新安名医堂品牌进一步打响。落实全面二孩政策，人口自然增长率5.41‰。成功举办全民健身运动会、市运会暨残运会、中国黄山国际登山大会、国际山地车公开赛,黄山籍著名运动员吕秀芝在里约奥运会等重大赛事上取得佳绩，我市获批国家体育产业基地。全面落实安全生产责任制，扎实开展“铸安”行动，连续14年荣获全省安全生产先进单位。成功创建省食品药品安全城市。“六五”普法全面完成，“七五”普法深入开展。圆满完成第九届村委会换届选举。加大矛盾纠纷排查化解力度，依法规范信访秩序，加强社会治安综合治理，强化应急体系建设，社会大局保持和谐稳定。军民融合深度发展，国防教育、国防动员、后备力量建设和军事设施保护、人民防空工作不断加强，休宁籍“兵王”王忠心荣获“八一勋章”，我市荣获全国双拥模范城四连冠、全省双拥模范城六连冠。妇女、未成年人、老年人、残疾人权益得到保障。哲学社会科学、新闻出版、广播电视、文学艺术、统计、地方志等工作得到加强。气象、档案、地震、防灾减灾工作取得新进展，民族宗教、外事侨务、对台等工作取得新成绩。

**七是坚持不懈提效能、优服务，自身建设成效显著。**认真开展党的群众路线教育实践活动、“三严三实”专题教育、“两学一做”学习教育、“讲看齐、见行动”学习讨论、“讲政治、重规矩、作表率”专题教育和专题警示教育，深入推进“两学一做”学习教育常态化制度化。扎实推进依法行政，健全完善重大事项依法决策机制，市政府相对集中行使行政复议权改革工作获“第四届中国法治政府奖”。加强政府立法工作，《黄山市徽州古建筑保护条例》经省人大常委会审查通过，提请市人大常委会审议《黄山市松材线虫病防治条例》，《黄山市城市市容和环境卫生管理规定》等3部规章颁布施行。自觉接受市人大及其常委会的法律监督、工作监督和市政协的民主监督，人大代表建议和政协提案全部办结。深入开展机关效能建设，组织实施政府提速和政府服务创新工程，建立“五位一体”政风评议和效能考评结果运用新机制。严格执行中央八项规定精神和省委、市委有关要求，全市“三公”经费支出比2012年下降54.5%。加强政务服务中心标准化建设，建立领导干部坐班政务服务中心制度，加快推行“最多跑一次”改革，所有行政审批项目实现“一门受理、一门发证”，提前办结率达96.3%。“12345”统一呼叫中心上线运行，累计办理有效群众诉求5.7万余件，满意率达94%。深化政务公开和政府信息公开，主动接受社会公众和新闻舆论监督。认真落实党风廉政建设主体责任和“一岗双责”，深入推进政府系统党风廉政建设和反腐败斗争，在全省率先开展“两个责任”痕迹管理，全面加强廉政风险防控，强化行政监察和审计监督，深入开展侵害群众利益的不正之风和腐败问题专项整治，严肃查处了一批违纪违法案件。

各位代表！

刚刚过去的2017年，是党的十九大召开之年，是供给侧结构性改革的深化之年，也是本届政府任期的最后一年。一年来，我们全力以赴稳增长，坚定不移促改革，持之以恒调结构，统筹城乡抓建设，深耕厚植强优势，尽心竭力惠民生，经济社会发展保持良好势头，主要指标稳中向好、全面增长。初步测算，地区生产总值645亿元，增长7.8%；财政收入106亿元，增长7%；固定资产投资645.5亿元，增长8%；社会消费品零售总额350.6亿元，增长12%；规上工业增加值增长10%；外贸进出口总额7.15亿美元，增长10%；旅游接待量5780万人次，增长11.4%，旅游总收入506亿元，增长12.4%；城镇和农村常住居民人均可支配收入分别增长8.5%和9%，较好完成了市六届人大七次会议确定的各项目标任务，顺利实现了本届政府的圆满收官。

各位代表！

五年来的不懈奋斗，奠定了全面建成小康社会、创造更加美好生活的坚实基础，积累了应对复杂环境、战胜风险挑战的宝贵经验，开启了践行新理念、推动新发展的重要阶段。这些发展成绩，得益于省委、省政府和市委的坚强领导，得益于市人大、市政协的监督支持，得益于全市广大干部群众的共同奋斗。在此，我代表市人民政府，向全市人民，向各民主党派、各人民团体和各界人士，向驻黄解放军指战员、武警官兵和政法公安干警，向关心、支持黄山发展的中央、省驻黄单位和海内外友好人士，向在我市创业的投资者、建设者，表示衷心的感谢!

在肯定成绩的同时，我们也清醒看到，我市发展不平衡不充分的问题依然突出。经济总量规模不大，农村一二三产业融合发展不够，新兴工业基础较弱，服务业发展不优，生态文化优势尚未有效转化为产业优势，加快构建现代产业体系任重道远；牵动性强的大项目尤其是产业类项目不多，实体经济困难较多，企业自主创新能力不强，财政收入质量不优，发展质量效益有待进一步提高；基础设施建设仍有欠账，城乡居民收入增长后劲不足，稳定脱贫任务繁重，就业、教育、社保、医疗、养老、居住、环境等民生领域还存在不少短板，人民群众还有不少操心事、烦心事；政府自身建设上，运用新发展理念创新性开展工作的能力和水平还不高，工作效能和效率有待进一步提升，政策落实“最后一公里”不够畅通，庸懒散拖等现象不同程度存在，一些领域腐败现象还时有发生。对此，我们一定高度重视，采取更加有力措施认真加以解决，不辜负各位代表和全市人民的期望和重托。

**迈进新时代，阔步新征程——今后五年目标任务**

党的十九大，开启了新时代全面建设社会主义现代化国家的新征程。新时代蕴含着新机遇，我国经济走向更高形态、更高质量，长期向好的基本面没有改变；大众旅游蓬勃兴起，国家和省把旅游业作为新常态下稳增长的新支点和重要支柱产业来培育，黄山发展迎来新的良机；绿色发展深入人心，生态文明建设体制机制创新加快，我市绿色生态优势必将加速向经济优势、竞争优势转变；中央把文化自信提到前所未有的高度，作为优秀传统文化的活态传承，徽州文化影响力持续扩大，必将成为支撑发展的重要软实力；我市现代综合交通体系日益完善，与长三角、京津冀、珠三角等城市群的时空距离大幅拉近，必将在迎接辐射中获取更多动能。新征程面临着新挑战，黄山的发展还累积不少深层次矛盾和问题，加之区域发展竞争日趋激烈，稍有松懈就会落后甚至掉队。我们必须坚定信心决心，抢抓发展机遇，沉着应对挑战，凝心聚力，开拓进取，奋力谱写新时代黄山发展的崭新篇章。

**今后五年政府工作的总体要求是：**紧密团结在以习近平同志为核心的党中央周围，高举中国特色社会主义伟大旗帜，以习近平新时代中国特色社会主义思想为指导，全面贯彻落实党的十九大精神，深入贯彻落实习近平总书记视察安徽重要讲话精神，按照省委十届六次全会和市委六届五次全会的部署要求，坚持稳中求进工作总基调，坚持新发展理念，紧扣社会主要矛盾变化，按照高质量发展的要求，统筹推进“五位一体”总体布局和协调推进“四个全面”战略布局，坚持以供给侧结构性改革为主线，坚持以五大发展行动计划为总抓手，坚持推进新型工业化和“旅游+”，坚持以人民为中心的发展思想，统筹推进稳增长、促改革、调结构、惠民生、防风险各项工作，在打好防范化解重大风险、精准脱贫、污染防治的攻坚战方面取得扎实进展，推动经济社会持续健康发展，加快建设更美丽更富裕更文明的现代化新黄山。

**主要目标是：**到2020年，与全国全省同步全面建成小康社会，“十三五”规划目标全面完成，市第六次党代会确定的目标任务顺利推进，全市生产总值达到850亿元，人均主要经济指标走在全省前列、主要社会生态指标走在全国前列，坚决打好防范化解重大风险、精准脱贫、污染防治攻坚战，确保全面建成小康社会得到人民认可、经得起历史检验。到2022年，力争全市生产总值突破1000亿元，城镇、农村常住居民人均可支配收入增速与经济增长保持同步，加快建设更美丽更富裕更文明的现代化新黄山，努力建成美丽中国先行区、世界一流旅游目的地和中国优秀传统文化传承创新区。

**主要任务是：**

**（一）突出创新驱动、科技支撑，构建现代产业新体系。**坚持质量第一、效益优先，深入开展创新型黄山建设，扎实推进供给侧结构性改革，实施大数据战略，推动经济发展质量变革、效率变革、动力变革，加快建设实体经济、科技创新、现代金融、人力资源协同发展的产业体系，使经济发展更具活力、更有韧性，推动经济在实现高质量发展上不断取得新进展。**加快建设技术创新体系。**深化科技体制改革，加快以科技创新为核心的全面创新，实施创新企业百强工程，加快建设国家和省级技术创新中心，强化知识产权创造、保护、运用，打造一批引领产业高端发展的骨干企业和具有创新潜力的“种子”企业，加快建立以企业为主体、市场为导向、产学研深度融合的技术创新体系。力争到2022年，院士工作站达10家，国家高新技术企业超过120家。**构建创新型现代产业体系。**强力推进新型工业化，以《黄山市工业转型升级规划》为引领，推动互联网、大数据、人工智能与实体经济深度融合，深入实施“三个百亿”计划，大力发展绿色食品、汽车电子、绿色软包装、新材料等主导产业，加快战略性新兴产业集聚发展和传统优势产业优化提升，规上工业增加值年均增长8%，战略性新兴产业产值年均增长15%，新增3个以上产值超百亿园区，形成5个以上百亿产业集群，打造支撑发展的“工业脊梁”。深入实施“旅游+”战略，推进全域旅游业态创新、综合管理体制创新和标准化建设，加快皖南国际文化旅游示范区建设，全面打响“中国旅游，黄山再出发”“梦幻黄山·礼仪徽州”品牌，游客接待量9000万人次、旅游总收入900亿元，过夜登记游客1200万人。大力发展现代服务业，促进生产性服务业专业化，推动生活性服务业个性化定制和融合发展，拓展互联网产业体系和网络经济空间，壮大现代金融、现代物流、文化创意、研发设计、现代商贸、健康养老、电子商务等产业规模，全市服务业增加值达540亿元、占全市生产总值比重提高2个百分点以上，把黄山现代服务业产业园建成全省领先、全国一流的文化旅游特色产业集聚发展基地。加快构建现代农业产业、生产和经营体系，积极探索小产区发展模式，促进农村三次产业融合发展，培育亿元以上农业产业化龙头企业50家、超10亿元产业集群8个、超50亿元产业集群2个，茶产业综合产值突破200亿元，建成休闲农业示范区10个。**打造创新创业人才高地。**深入实施高层次创新人才培养集聚、技能人才梯次培养发展、优秀企业家培育工程，引进和培育高层次科技人才团队10个、高层次人才150名，培养高技能人才2.5万人，跻身全省人才强市行列。

**（二）突出区域协调、乡村振兴，形成城乡融合发展新格局。**深入开展全国“城市双修”试点，扎实推进全国文明城市等“六城联创”和全域环境整治，加速打造现代化区域性中心城市，力争到2022年，户籍人口城镇化率达到37.5%。**提升中心城区城市功能。**统筹推进“一环三片”建设，加快构建三江口核心商圈，规划建设以“城市绿核”为依托的城市森林公园，加快道路、桥梁、停车场、“三供”、地下管廊、园林绿化、市容环卫等基础设施和教育、文化、卫生、体育等公共服务设施建设，提升中心城区人口集聚度和综合承载力，建成区面积超过66平方公里。以中心城区为核心，加快市域南部城镇群同城化步伐，实现城市基础设施、公共服务设施、安全保障设施共建共享、互联互通。**加快县域经济发展。**强化各区县主体功能定位，以产业创新为基点，加大对县域新兴产业、特色产业发展的支持力度，加快推动县域经济新旧动能转换，力争地区生产总值超过百亿元的县（区）达到6个。加快县域重大基础设施建设，完善城市功能，提升服务效能，让更多的要素向县域集中，形成特色取胜、错位发展的良好格局。**推进特色小镇建设。**以国际化视野定位小镇、特色文化引领小镇、特色产业发展小镇、市场机制建设小镇、改革理念激活小镇，建成50个左右产业“特而强”、 功能“聚而合”、形态“小而美”、机制“新而活”的特色小镇，国家和省级特色小镇总数达到15个以上，培育一批有影响力的小镇品牌，形成具有竞争优势的特色小镇集群，加快打造经济增长新引擎、新型城镇化建设新平台。**实施乡村振兴战略。**坚持农业农村优先发展，按照产业兴旺、生态宜居、乡风文明、治理有效、生活富裕的总要求，建立健全城乡融合发展体制机制，完善乡村治理体系，推进农业农村现代化与城镇化、工业化、信息化“四化同步”发展。打造美丽乡村“升级版”，引进优质资本、优势企业、优秀人才参与乡村开发，因地制宜发展乡村产业，积聚乡村人气，深入实施“三大革命”，加强农村基层基础工作，全面完成美丽乡镇建设，全市所有布点中心村达到美丽乡村建设要求、自然村环境整治全覆盖，美丽乡村建设继续走在全省前列。

**（三）突出绿色生态、宁静和谐，建设美丽宜居新家园。**牢固树立和践行绿水青山就是金山银山的理念，积极探索绿水青山向金山银山有效转化的路径，加快形成节约资源和保护环境的空间格局、产业结构、生产方式、生活方式，着力打造生态文明建设安徽样板的示范先行区。**加快转变发展方式。**落实国家主体功能区战略，加快生态文明先行示范区建设，推进大黄山国家公园创建，加快建设山水相济、人文共美的新安江生态经济示范区，努力构建绿色低碳循环发展的产业体系。推进资源全面节约和循环利用，确保万元生产总值能耗、主要污染物排放总量控制在省下达指标内。倡导简约适度、绿色低碳的生活方式，推进国家低碳城市试点，让尊重自然、顺应自然、保护自然成为全市人民的思想共识和行动自觉。**着力解决突出环境问题。**加快构建政府为主导、企业为主体、社会组织和公众共同参与的环境治理体系，严格落实“水十条”“土十条”，深入开展环境保护“五个一”专项行动，坚决打好大气、水、土壤污染防治攻坚战，确保森林覆盖率、水环境、土壤环境和空气质量继续保持全国领先。**切实加大生态系统保护力度。**深入实施新安江流域综合治理，积极构建市场化、多元化的生态补偿长效机制。落实最严格的环境保护制度和生态环境损害责任终身追究制，严守生态保护红线、永久基本农田红线、城镇开发边界，统筹山水林田湖草系统治理，提供更多优质生态产品，让黄山的河流清澈如镜、大地绿草如茵、山坡森林如屏、空气清新如洗。

**（四）突出开放融合、内外联动，打造内陆开放新高地。**坚持引进来和走出去并重，实行更加积极主动的开放战略。**拓展开放发展新空间。**主动参与“一带一路”建设，拓展与国际组织、国际友城合作。积极融入长三角一体化和长江经济带，主动对接京津冀、珠三角、海西经济区发展，充分发挥新安江生态经济示范区效应，推动产业融合、政策协同，加快融入杭州都市圈，形成双向互动、内外联动的全面开放新格局。**构建对外开放大通道。**全面推进以“一港两轨八铁九路”为主干的现代综合交通体系建设，加快将黄山建设成全国高铁“八纵八横”主骨干网络的重要节点、国家公路运输枢纽、国际空港城和皖浙赣区域综合交通枢纽。**增强外贸竞争新优势。**加强农产品、纺织服装、机械电子、新材料等出口基地建设，大力推进跨境电子商务发展，加快打造全国重要的茶叶出口基地，力争到2022年，全市进出口规模超千万美元企业20家、超5000万美元企业2-3家，五年累计外贸进出口总额突破40亿美元。**提升招商引资新水平。**创新招商模式，持续推进以商招商、产业链招商和小分队招商，力争五年累计引进亿元以上项目超600个、到位资金年均增长10%以上，新批外商投资企业30家，外商直接投资10亿美元。

**（五）突出共治共建、补齐短板，共享幸福美好新生活。**坚持以人民为中心，坚持在发展中保障和改善民生，多谋民生之利、多解民生之忧。**坚决打赢脱贫攻坚战。**坚持精准扶贫、精准脱贫，注重扶贫同扶志、扶智相结合，深入推进脱贫攻坚“十大工程”，确保到2020年，现行标准下农村贫困人口全部脱贫，做到脱真贫、真脱贫，确保全面建成小康社会一个不能少、共同富裕路上一个不掉队。**全面提升就业和社会保障水平。**坚持就业优先战略和积极就业政策，五年城镇新增就业10万人，城镇登记失业率控制在4.2%以内。全面建成覆盖全民、城乡统筹、权责清晰、保障适度、可持续的多层次社会保障体系，基本养老保险参保率达到90%以上，基本医疗保险参保率稳定在95%以上。实现城镇常住人口住房保障全覆盖。**推进基本公共服务均等化。**优先发展教育事业，深化教育改革，推进城乡义务教育一体化和优质均衡发展，扩大学前教育资源，普及高中阶段教育，完善现代职业教育体系，加快教育现代化，办好人民满意的教育。深化医药卫生体制改革，传承发展新安医学，加快建设健康黄山。广泛开展群众体育活动，打造运动休闲健康城市。培育和践行社会主义核心价值观，健全完善公共文化服务体系，繁荣兴盛徽州文化。**建设更高水平的平安黄山。**加快构建共建共治共享的社会治理格局，推进数字黄山和智慧黄山建设，健全公共安全体系，确保社会大局和谐稳定，让全市人民的获得感、幸福感、安全感更加充实更有保障更可持续。

各位代表！功崇惟志，业广惟勤。我们坚信，幸福都是奋斗出来的，只要全市上下勠力同心、奋勇拼搏，古老的徽州大地必将谱写出新时代的华彩篇章，一个更美丽更富裕更文明的现代化新黄山必将展现在世界面前！

**坚实起步，扎实开局——2018年主要工作**

今年是全面贯彻党的十九大精神的开局之年，是改革开放40周年，是决胜全面建成小康社会、实施“十三五”规划承上启下的关键一年，做好今年各项工作，意义重大。经济社会发展的主要预期目标是：全市生产总值增长7.5%以上，财政收入增长7%，固定资产投资增长8%，社会消费品零售总额增长10%，城镇常住居民人均可支配收入增长8.5%，农村常住居民人均可支配收入增长9%，居民消费价格涨幅3%左右，万元生产总值能耗、主要污染物排放总量控制在省政府下达目标以内。

**重点做好以下八个方面工作：**

**（一）全力促进经济平稳健康发展**

扎实推进供给侧结构性改革，深入实施五大发展行动计划，全面落实市促进重点产业发展“1+N”政策，着力增强经济增长动力和活力。

**深入推进供给侧结构性改革。**继续抓好“三去一降一补”，重点在“破”“立”“降”上下功夫。优化存量资源配置，扩大优质增量供给，分类处置“僵尸企业”，加快淘汰落后产能。大力培育新动能，深入实施创新驱动发展战略，加快技术和产业、平台和企业、金融和资本、制度和政策“四大创新”体系建设，推动传统产业优化升级，培育一批具有创新能力的排头兵企业。大力降低实体经济成本，全面落实国家和省市减税降费政策，继续清理涉企收费，持续降低企业制度性交易成本和融资、用工、用能、物流、土地使用成本。

**持续扩大有效投入。**全力抓好重点项目建设，力争新开工100个以上、竣工70个以上市级重点项目，确保完成重点项目投资300亿元以上。基础设施方面，加快推进芜黄高速、新安江街口大桥、500千伏徽州变升压增容工程等项目，开工建设池黄高铁、昌景黄高铁、黄千高速、德上高速，积极推进杭临黄高铁、黄衢高铁、长陔岭隧道项目前期工作，确保杭黄高铁、歙黟一级公路等建成运营，力争屯溪国际机场迁建纳入国家民航“十三五”规划中期调整。产业发展方面，加快推进未来科技城、小罐茶总部基地、联合利华立顿茶叶等项目，开工建设无源芯片主题产业园、顺威智能家居等项目，确保博蓝特半导体、神剑新材料、天之都环保科技、九龙科创园等建成运营。围绕国家政策导向和中央预算内资金投向，调整完善五大发展行动计划项目库，深度谋划亿元以上项目100个，力争更多项目进入国家和省年度投资计划。完善促进民间投资支持政策，鼓励社会资本参与基础设施和公共服务领域建设。严格落实“四督四保”和“五项机制”，建立健全重大项目服务、调度和督查机制。

**强化金融支撑保障。**创新金融产品、优化信贷结构，加大服务实体经济特别是中小微企业力度，力争金融机构贷款余额超750亿元。用足用活“4321”新型政银担、税融通政策，充分发挥续贷过桥资金作用。加快多层次资本市场体系建设，支持黄山旅游、永新股份等上市公司实施再融资和并购重组，推进昌辉股份（黄山）、泰达新材料等企业主板首发上市，鼓励企业在“新三板”、省区域性股权市场挂牌融资，支持安元现代服务业基金、黄山赛富基金积极运作，积极引进保险直投资金，力争全年新增“新三板”挂牌企业2家以上、省股权托管交易中心挂牌企业16家、上市挂牌后备资源企业20家，直接融资30亿元以上。大力发展普惠金融，稳妥推进农村资金互助，抓好黄山区全国农村承包土地经营权抵押贷款试点。加强金融精准扶贫，用好再贴现、再贷款政策，提高扶贫小额贷款资金使用效益。加强社会信用体系建设，强化影子银行、互联网金融、非法集资等风险防范和处置，坚决打击违法违规金融活动。

**（二）强力推进新型工业化**

牢固树立“工业强市、产业兴市”理念，聚焦主导产业，聚力园区建设，深入推进“三重一创”建设，做大总量、提升质量，力争规上工业增加值增长10%。

**培育壮大主导产业。**加强工业经济运行监测，深入实施小微企业成长计划，发挥市级队企业“头雁效应”，力争市级队企业产值增速高于规上工业2个百分点以上。突出抓好绿色食品、汽车电子、绿色软包装、新材料等四大主导产业集群集聚发展。组织实施天然饮用水产业发展规划，力争实现饮用水产业招商引资新突破。抓好文化旅游省级战略性新兴产业集聚发展基地特色化发展，启动2个市级战略性新兴产业集聚发展基地建设，确保全市战略性新兴产业产值增长15%以上。

**加快开发区改革创新发展。**全面落实开发区改革和创新发展实施意见，力争全市开发区经营收入、规上工业增加值、固定资产投资均增长10%。加快“腾笼换鸟”，持续推进闲置低效土地清理处置。发挥市经济开发区在体制机制创新、发展质量和速度上的“头雁效应”，持续推进轩辕大道、迎客松大道和非遗创意园、菲英离合器、中鼎信息等项目建设，开工建设市开发区创新大厦，进一步拉开框架、优化发展布局、完善城市功能，积极争创国家级高新技术开发区，力争工业总产值增长18%、招商引资到位资金增长20%。全面完成全市开发区整合工作，推进歙县和徽州区循环经济园基础设施共建共享，支持歙县开发区申报国家级开发区，推动全市各开发区特色化、差异化发展。

**着力推进科技创新。**滚动实施100个重点技改项目，鼓励企业实施“零地技改”“机器换人”，力争工业投资和技改投资均增长10%。支持企业开展产学研用合作，新增省、市级创新平台8家以上，市级队企业、高新技术企业研发投入分别达到销售收入的2%、4.5%。深入开展“互联网+”制造行动，新增省级智能工厂和数字化车间2家。全力推进国家火炬黄山软包装新材料特色产业基地和4个省级高新技术产业基地“二次创业”，全年新增高新技术企业5家，高新技术产业增加值增幅高于规上工业增加值增幅2个百分点以上。

**持续开展“四送一服”双千工程。**坚持市县联动，进一步落实联系包保机制，主动作为，提升服务企业的针对性和实效性。建立完善服务实体经济网络平台，畅通企业问题受理渠道，健全限时反馈、跟踪督办、自动提醒等制度，切实解决企业生产经营中的实际困难和问题。

**（三）着力提升服务业发展水平**

围绕打造世界一流旅游目的地，深入实施“旅游+”发展战略，加快推进文化旅游国际化，努力形成多点支撑、多业融合的现代服务业发展格局。

**大力发展全域旅游。**深入实施皖南国际文化旅游示范区“1+5”行动计划，扎实推进国家级旅游业改革创新先行区建设，全面推动黄山旅游“品质革命”，力争游客接待量和入境游客分别增长11%和10.5%，旅游总收入增长13%，过夜登记游客超950万人。以国际化标准谋划大黄山国家公园建设，全面启动黄山东海景区索道、“通天坦”营地等项目，积极推进东黄山国际小镇市政基础设施和南组团核心区建设。开展西递宏村、古徽州文化旅游区等重点景区提质增效行动，启动牯牛降5A景区创建工作，力争齐云山5A景区、太平湖国家旅游度假区获批，高起点高水平启动月潭湖基础设施建设。实施花山谜窟提升工程，丰富屯溪老街、黎阳in巷、湖边古村落经营业态，规划“夜游新安江”提升工程。大力发展修学研学、摄影写生、户外运动、健康养生、宗族寻根之旅，开发自驾车房车营地、青少年户外运动营地、自然生态旅游区、低空飞行、亲子游等多元化、个性化、体验式新兴产品，推出徽州精品民宿50佳、规划打造民宿集群20处，推动观光旅游逐步向休闲度假旅游转变。创新办好黄山旅游节、国际登山大会、国际山地车公开赛、168国际徒步探险、国际乡村摄影大展等品牌赛事活动，持续扩大“梦幻黄山·礼仪徽州”城市品牌影响力。巩固长三角及省内近程市场，扩大高铁沿线等中程市场，开辟东北、西北、西南远程市场；巩固台湾和欧美、韩国、泰国等入境市场，恢复香港旅游包机，力争开通俄罗斯、日本、新加坡、马来西亚、柬埔寨等国包机。启动第二轮旅游厕所新三年行动计划，抓好高速公路服务区旅游化改造，完善全域旅游标准化体系，充分发挥旅游大数据中心作用，健全游客服务咨询体系，健全“1+3”旅游综合管理机制，推进旅游综合执法全覆盖，提升游客满意度，力争全域旅游示范区创建率先通过国家验收。

**加快发展现代服务业。**做大做强黄山现代服务业产业园，加快核心区“大三角”及向西延伸区域开发建设，强力推进文化旅游、新兴金融、高技术服务产业招商，加快建设中锐国际教育小镇、昌仁医养综合体、市文化艺术中心，力争徽菜博物馆、金融小镇竣工运营，基本建成黄山现代服务业产业园起步区。编制实施大健康产业规划，大力发展以新安医学为特色的健康养生产业，抓好“黄山贡菊”皖药基地建设，全面落实康美药业与我市战略合作协议，积极推动医旅结合、医养整合、多业融合。统筹推进省级服务业集聚区、示范园区和综合改革试点县建设，重点培育壮大50家服务业市级队企业，加强限上商贸流通企业培育，积极打造特色街区，大力发展文化创意、研发设计和互联网金融产业，确保服务业增加值增幅高于全市生产总值增幅。

**（四）全面实施乡村振兴战略**

深化农村综合改革，加快推进农业农村现代化，加大美丽乡村建设力度，打造农民幸福生活美好家园。

**深入推进农村改革。**全面开展农村集体产权制度改革，基本完成乡镇、村组集体资产清产核资工作，稳步推进农村经营性资产股份合作制改革。抓好黟县农村“三变”改革整县推进工作，有序开展其他区县试点工作。深化黟县多层次农业保险试点工作。发展壮大农村集体经济，健全完善农村集体“三资”管理监督和收益分配制度，更好保障农民财产权益。完善“三权”分置改革，落实集体所有权，稳定农户承包权，放活土地经营权。深化小型水利工程产权制度改革。巩固国有林场改革成效，探索开展集体林业股份化经营。依托江南林业产权交易所，基本建成市县乡三级农村产权交易服务体系。

**大力发展精致农业。**全力实施农业产业化“826”行动计划，扎实开展农业绿色增效行动，全年培育巩固年加工产值超10亿元产业集群2-3个，规模以上农产品加工企业产值增长10%。深入开展茶业全产业链有机化试点示范，改造低产低效茶园5万亩，建设高效生态茶园3万亩；办好黄山茶会，强化品牌营销，实现茶叶一产产值36亿元。稳定发展粮食生产，大力推广稻虾（蟹）混养模式。继续抓好20个重点区域油菜优质高产示范片创建，因地制宜发展干鲜果、食用菌、覆盆子、皖南花猪、黄山中蜂等特色种养业，山泉流水养鱼产业综合产值突破2亿元。扎实推进休宁县国家级有机产品认证示范区、祁门县国家级出口茶叶质量安全示范区创建，持续打造一批区域公共品牌，加快建立农产品质量可追溯体系，推出一批在全国叫得响的有机绿色农产品。

**推进农村一二三产业融合发展。**大力培育新型农业经营主体，加强农民专业合作社规范化建设，全年新发展家庭农场150个、现代农业产业化联合体15个。深入实施休闲农业与乡村旅游提升工程，继续办好中国休闲农业黄山峰会，加快十大休闲农业示范区建设，推进歙县蓝田·桃源国家级田园综合体试点，新打造10个左右精品休闲农庄。抓好电子商务产业集聚区建设，巩固提升电子商务进农村全覆盖工作成果，创建省级电商特色小镇8个以上、电商示范村20个以上，全年农产品电子商务交易额10亿元。

**加快特色小镇建设。**持续推进首批12个特色小镇建设，突出抓好100个重点项目，力争全年完成投资50亿元。启动第二批市级特色小镇建设，新增国家和省级特色小镇3个以上。突出抓好产业培育，建立“上海交通大学·黄山市特色小镇特色产业双创中心”。完善政策支持体系，健全动态调整、考核激励机制，探索黄山特色小镇发展新路。

**打造美丽乡村“升级版”。**持续推进2017年度42个乡镇政府驻地建成区整治、46个省级和20个市级中心村建设及自然村治理改造，确保通过省级验收。全面启动2018年度40个左右省级中心村、30个左右市级中心村建设和300个左右自然村治理改造。统筹推进农村“三大革命”和农村环境“六治理”，抓好“四好农村路”建设，加快实施世行贷款黄山新农村建设项目和传统村落保护项目。鼓励社会资本参与乡村开发，大力发展乡村特色产业，努力走出一条村美民富的美丽乡村建设可持续发展之路。

**（五）全面深化改革扩大开放**

以纪念改革开放40周年为契机，加强改革创新攻坚，持续扩大对外开放，切实增强发展动能，全面激发发展活力。

**推动重点领域改革。**大力发展智慧经济，统筹规划建设数据基础设施，推进数据资源共建共享，组建市大数据管理机构，加快建设数字黄山。深化“放管服”改革，加强权责清单动态管理，完善涉企收费清单、政务服务清单制度，推动公共服务清单和行政权力中介服务清单落地生效。深化行政审批制度改革，继续做好国务院、省政府取消下放行政审批事项的承接落实工作。全面推行“双随机一公开”跨部门联合检查，实现监管全覆盖。持续推进商事制度改革，加强“多证合一”运用，推进企业登记全程电子化。加快推进“互联网+政务服务”，建成覆盖全市的政务服务“一张网”，实现政务服务事项“应上尽上，全程在线”。深化财政体制改革，把握积极的财政政策，调整优化财政支出结构，确保对重点领域和项目的支持力度，加强优质长效税源培植，做实做优财政综合实力，压缩一般性支出，切实加强政府债务管理。深化价格机制改革，完善重点领域价格形成机制。全面启动公立医院编制周转池试点，加快建立现代医院管理制度和人事薪酬制度，全面推进医联体、县域医共体建设。深化事业单位分类改革，有序推进市直事业单位“撤并转”。深化国资国企改革，推进黄山旅游集团“二次创业”，组建市交投集团，推动城投、文投、信投、供销集团建立现代企业制度，做大做强主业。依法保护各种所有制经济产权和合法利益，启动实施市场准入负面清单制度，加快构建亲清新型政商关系，着力创优“四最”营商环境，进一步激发和保护企业家精神，深入推进大众创业万众创新，加快建设一批低成本、便利化、全要素、开放式众创空间，新增市场主体9600户。

**打造内陆开放新高地。**把招商引资作为重中之重，坚持引资和引智引技相结合，全面深化与北京市国资委、上海市国资委的合作，推进与北京城建、北京工美、上海城投、光明集团等一批合作项目落地开工。加大与央企的合作，力促中信集团、中交集团等一批合作项目早日签约。组建“产业+”招商小分队，健全“三个清单”招商方式，力争引进世界500强、国内500强取得新突破。全年新签亿元以上项目超120个，实际到位资金增长10%。加强与“一带一路”沿线国家和地区在茶产业、旅游、文化、教育等领域的国际交流合作，力争对“一带一路”沿线国家和地区的进出口额突破4亿美元。抓好进境水果口岸建设。依托口岸、保税仓库、开发区和重大展会，加快打造一批具有影响力和承载力的投资促进、贸易合作和对外交往新平台。加快国际贸易“单一窗口”建设，巩固机电、新材料、纺织服装和农产品出口市场，扩大高新技术产品出口份额，引导企业增加先进设备、关键零部件、重要原材料进口，大力推进跨境电子商务发展，全年外贸进出口总额增长7%。

**（六）切实抓好城市建设**

坚持以新型城镇化为引领，扎实推进全国“城市双修”试点和“两治三改”三年专项行动，围绕“六城联创”，全面提升数字化、精细化建设管理水平，加快打造现代化区域性中心城市。

**抓好中心城区和区县城建设。**围绕中心城区“一环三片”，突出抓好路网建设，开工建设新安月桥、八一桥、黎阳桥、阳湖桥，实施30条道路改造提升工程。加快屯溪老街滨江西路综合提升、花溪饭店地块改造、外边溪开发，推进“徽州坊”文化旅游综合体建设，打造三江口核心商圈。积极创建省级优先发展公共交通示范城市，建设一批公共停车场；抓好海绵城市建设，加快雨污分流和污水收集全覆盖，推进排水防涝三年行动，实施一批城市园林精品工程；开工建设市资源循环利用基地，建成运营市生活垃圾综合处理厂（一期），开工建设市三水厂，推进市域南部城镇群水务一体化。加快屯、徽、休、歙同城步伐，大力实施区县城老城改造和新城建设，有序推进农村转移人口市民化，力争户籍人口城镇化率提高1.3个百分点。

**加强城乡规划管理。**完成市县两级空间规划数据库建设，编制智慧城市、传统村落保护利用示范区、历史文化名城名镇名村保护、城市步行和自行车交通系统建设规划。深化城管执法体制改革，整合城市供排水、燃气、污泥处置等管理职能，集中行使部分行政处罚权及行政强制权，推进城市管理数字化、智慧化。全面实施三横四纵三区域建筑立面风貌整治和亮化提升。推进美丽社区建设“七大工程”，扎实做好社区协同治理工作，健全老旧小区物业管理机制。加强社会主义核心价值观教育，提升市民精神文明素质。

**（七）持续厚植生态文化优势**

坚持保护与利用并举、传承与创新并重，持续加强生态文明建设，推动徽州文化创造性转化、创新性发展，着力打造核心竞争力、增强文化软实力。

**加大生态保护力度。**全面完成中央和省环保督察问题整改落实，加大生态环境保护执法力度。巩固新安江流域生态补偿机制二轮试点成果，实施新安江生态经济示范区规划，用好用活新安江绿色发展基金。举办好第16届中国水论坛。实行“一河一策”，全面推进河（湖）长制管理，加大非法排污企业巡查整治力度。加快月潭水库建设，全年完成投资10亿元。加快太平湖国家生态保护专项、阊江流域治理建设。继续实施中小河流治理、水土保持等项目，争取实施山水林田湖草工程。继续推进农药集中配送工作，实施化肥使用量零增长行动。建成运营农村生活垃圾收运系统，加快建设农村污水治理项目（一期）。推进林业增绿增效行动，实施林长制，全年封山育林8.5万亩，森林抚育58.8万亩。强化林业有害生物防治，力争实施松材线虫病防治三期工程，确保黄山松安全。严格落实耕地保护主体责任，大力推进补充耕地建设，确保全市耕地保有量和基本农田保护面积不减少。加强地质灾害防治，完成30处隐患点治理。严格落实节能减排目标责任制，加强固体废弃物处置，抓好秸秆禁烧和综合利用、城区扬尘污染防治，全力打赢蓝天保卫战。

**繁荣兴盛徽州文化。**坚持徽文化活化利用，挖掘徽文化道德内核，借鉴徽文化乡贤乡约，加强徽文化保护传承，发挥徽文化时代价值，努力走出一条保护与利用良性互动的新路子。扎实开展历史建筑保护利用试点，加快推进168个徽州古建筑保护利用工程项目建设，完成投资5.8亿元。启动皖南古村落世界遗产拓展项目申报，支持徽州古城城墙申报世界文化遗产，力争黟县获批国家历史文化名城，一批古村落列入中国传统村落名录。深入推进徽州文化生态保护实验区建设，实施徽州文化经典数字化工程和徽剧振兴工程，加快设立徽州书院，修缮一批名人故居、古祠堂，建好一批村史馆、乡贤馆，编撰出版《徽州文物谱系》。深化与故宫博物院战略合作，故宫文创馆徽派传统工艺馆开馆运营。完善非物质文化遗产数据库。推进名镇名村名街志和特色志编纂工作。开展徽州四雕、徽墨歙砚、万安罗盘、徽州漆器等保护性开发，抓好国家级非遗项目、传习基地和传承人队伍建设。繁荣文艺创作，力争1-2个项目入围国家艺术基金。完善公共文化服务体系，实施国家级徽州民间文艺展演示范项目，深入推进文化惠民工程，力争市图书馆跻身国家一级馆。挖掘优秀徽州传统文化，积极打造徽州IP和文化热点，讲好黄山故事，开发衍生产品，做活转化文章。加快文化产业集聚区建设，支持屯溪老街文化产业基地做大做强，大力培育文化知名企业、新型业态，抓好100个重点文化产业项目建设，力争完成投资80亿元，争创省级以上文化产业示范基地5家以上，力争文化产业增加值增长10%以上。

**（八）提高保障和改善民生水平**

以造福人民为最大政绩，想群众之所想，急群众之所急，让人民生活更加幸福美满。

**坚决打赢脱贫攻坚战。**坚持精准扶贫精准脱贫基本方略，巩固脱贫成果，提高脱贫质量。扎实开展扶贫领域作风问题专项整治，构建扶贫领域作风建设长效机制。强化基础工作，加强动态调整和程序管控，建立精准扶贫大数据管理平台，完善贫困监测体系，确保对象精准、信息准确。狠抓政策落地，按照“五个一批”基本路径，深入实施脱贫攻坚“十大工程”。坚持产业扶贫优先，坚持扶贫与扶志、扶智相结合，激发贫困人口内生脱贫动力；加强基层基本公共服务功能建设，认真落实“351”“180”等政策，加快构建覆盖所有贫困人口的医疗保险和救助制度，实现建档立卡贫困学生教育资助全覆盖；提前完成“十三五”易地扶贫搬迁任务。多渠道发展壮大村集体经济，夯实稳定脱贫基础。深入开展“万名党员干部帮万户”“百企帮百村”，巩固153个出列村脱贫成果，实现12354人脱贫目标，力争脱贫攻坚工作位列全省第一方阵。

**持续加强民生保障。**认真组织实施省市民生工程，健全常态长效管养机制，确保民生工程工作走在全省前列。加强就业培训和创业服务，全年城镇新增就业2万人，城镇登记失业率控制在4.2%以内。全面实施全民参保计划，落实企业和机关事业单位退休人员基本养老金调整政策。提高新农合、城镇居民医保、基本公共卫生服务补助标准，做好最低生活保障和特困人员救助供养提标工作，继续实施困难残疾人生活补贴、重度残疾人护理补贴制度。支持社会力量兴办养老服务机构，推动居家养老、社区养老、机构养老衔接发展。加快城镇棚户区改造和老旧小区整治，完成1329套城镇棚户区改造和51个老旧小区整治任务。健全促进房地产市场平稳健康发展长效机制，扩大公租房保障范围，加快建立多主体供给、多渠道保障、租购并举的住房制度。

**大力发展社会事业。**深入实施人才强市战略，认真落实“人才10条”，引进高层次人才30名，新增省级博士后工作站1家。推进第三期学前教育行动计划，启动义务教育优质均衡试点，理顺中心城区义务教育管理体制，深化普通高中课程改革，强化职业教育市级统筹，全面实施中小学教师“无校籍管理”，尤其要着力解决中小学生课外负担重、“大班额”等问题，解决好婴幼儿照护和儿童早期教育服务问题。支持黄山学院建设地方性应用型高水平大学、黄山电大建设开放大学。力争市委党校新校区秋季开班。深入实施全民科学素质行动计划纲要，不断提高全民科学素质。推进健康黄山建设，继续解决好“看病难、看病贵”问题，鼓励社会资金进入养老、医疗等领域，实施新安医学振兴工程，深入开展爱国卫生运动，抓好重大疾病防控，提高家庭医生履约率和履约水平，加快人口健康信息平台建设，提升公共医疗管理和健康服务水平。全面落实计划生育各项政策，加大计生困难家庭帮扶力度。广泛开展全民健身运动，积极备战省十四届运动会和省七届残运会。启动第四次全国经济普查。充分发挥工会、共青团、妇联等人民团体的重要作用。健全农村留守儿童和妇女、老年人、残疾人关爱体系。做好民族、宗教、物价、档案、地方志等工作。推进军民融合深度发展，加强国防教育、国防动员、后备力量建设和军事设施保护、人民防空工作，支持部队改革和停偿工作，做好优抚安置工作，深入开展全国、全省双拥模范城（县）创建。

**加强和创新社会治理。**发挥社会服务管理信息化平台作用，建设统一政府热线服务平台，提高网格化管理和一站式服务水平。加强基层组织建设，完成第十届村委会换届选举和城市社区居委会第四次换届选举工作。深入开展“七五”普法，做好人民调解、法律援助和社区矫正、安置帮教等工作。健全矛盾纠纷多元化解机制，依法规范信访秩序，启用新市民接访中心，加快各级综治中心标准化建设，努力把矛盾纠纷解决在基层、化解在萌芽状态。全面落实安全生产责任制，深入开展“铸安”行动，突出抓好重点行业领域安全专项治理，严格排查和整治各类安全隐患，确保安全生产形势持续稳定好转。加强应急管理，做好防汛抗旱、森林防火和气象、地震等工作，提升防灾减灾救灾能力。加大食品药品安全监管力度，积极争创国家食品安全示范城市。加强社会治安防控体系建设，推进公共安全视频“雪亮工程”建设，依法打击和惩治各类违法犯罪活动，确保社会大局和谐稳定。

**不忘初心，牢记使命——建设人民满意的服务型政府**

新时代开启新征程,新时代呼唤新作为。各级政府及其工作人员要全面增强学习、政治领导、改革创新、科学发展、依法执政、群众工作、狠抓落实、驾驭风险八种本领，初心不改、矢志不渝，善作善成、一往无前，坚定不移干在实处，走在前列。

**坚定政治信仰，全力建设忠诚政府。**坚持党对一切工作的领导，把政府系统党的政治建设摆在首位，深入推进“两学一做”学习教育常态化制度化，扎实开展“不忘初心、牢记使命”主题教育，牢固树立“四个意识”，坚定不移忠诚维护看齐习近平总书记这个党的领袖，坚决维护习近平总书记在党中央和全党的核心地位，在政治立场、政治方向、政治原则、政治道路上同以习近平同志为核心的党中央保持高度一致。坚持在全局下定位、大局下行动，认真贯彻落实省委、省政府和市委各项重大部署，确保政令畅通、执行有力。严明党的政治纪律和政治规矩，严格执行《新形势下党内政治生活若干准则》《中国共产党党内监督条例》，认真贯彻民主集中制，自觉弘扬和践行党内先进政治文化，努力营造风清气正的良好政治生态。

**坚持依法行政，全力建设法治政府。**全面落实法治政府建设实施纲要，严格依照法定权限和程序履行职能。健全重大决策征询意见、听证、风险评估、合法性审查、实施效果评估、责任追究等机制，建立完善行政决策专家论证制度，加强重大事项决策全过程痕迹管理。健全科学民主依法立法机制，建立立法专家咨询制度，制定出台《黄山市河（湖）长暂行规定》《黄山市美丽乡村建设促进暂行办法》，起草《黄山市农药集中配送条例》。推行行政执法公示、执法全过程记录、重大执法决定法制审核“三项制度”，严格规范公正文明执法。探索建立行政复议案件庭审制度。抓好徽州区全国基层政务公开标准化规范化试点，全面推进决策、执行、管理、服务、结果“五公开”。落实重大决策和重大项目向人大及其常委会报告制度，加强与政协民主协商，广泛听取各民主党派、工商联和无党派人士意见，自觉接受人大及其常委会的法律监督、工作监督和政协的民主监督，严格落实人大及其常委会的决议决定和审议意见，认真办理人大代表议案、建议和政协提案，提高办理质量和满意度。

**强化使命担当，全力建设效能政府。**持续抓好机关效能建设，深入推进政府服务创新，组织实施政府服务提质提效工程，完善网上办事大厅服务功能，加快推进“最多跑一次”和“不见面审批”改革，努力为人民群众和各类市场主体提供更便捷、更周到、更高效服务。完善抓落实的制度体系，健全全面覆盖的督查机制，加大问责追责的力度，确保各项决策部署掷地有声、落地见效。坚持严管和厚爱结合、激励和约束并重，建立健全激励机制和容错纠错机制，旗帜鲜明地为敢于担当、踏实做事、不谋私利的干部撑腰鼓劲，充分调动广大干部的积极性、主动性、创造性。

**加强反腐斗争，全力建设廉洁政府。**严格落实《中国共产党廉洁自律准则》《中国共产党纪律处分条例》，认真履行党风廉政建设主体责任和“一岗双责”，深入开展政府系统党风廉政建设和反腐败斗争。严格执行中央八项规定实施细则精神和省委、市委有关要求，认真查摆“四风”新表现，驰而不息狠抓作风建设。加强“两个责任”痕迹管理，强化对重点领域和关键环节权力运行制约监督，进一步规范项目招投标、土地使用权与矿业权出让、国有产权交易、政府采购等行为，实现公共资源“网下无交易、网上全公开”。健全廉政风险防控机制，强化财政资金监管和审计监督。坚持无禁区、全覆盖、零容忍，保持惩治腐败高压态势，加大整治群众身边腐败问题的力度，切实做到干部清正、政府清廉、政治清明。

各位代表！

潮平岸阔催人进，风劲扬帆正当时。让我们紧密团结在以习近平同志为核心的党中央周围，在省委、省政府和市委的坚强领导下，不驰于空想，不骛于虚声，锐意进取、埋头苦干，为加快建设更美丽更富裕更文明的现代化新黄山、谱写新时代中国特色社会主义的黄山篇章而努力奋斗！
